# Editorial: Graft-versus leukemia (GVL) effect - immunobiology and ways to augment it

**DOI:** 10.3389/fimmu.2024.1547194

**Published:** 2025-03-06

**Authors:** Mahasweta Gooptu

**Affiliations:** Department of Medical Oncology, Dana–Farber Cancer Institute, Boston, MA, United States

**Keywords:** GVL, immunology, relapse, post-transplant, adoptive immunotherapy

Relapse post allogeneic hematopoietic stem-cell transplantation remains the chief cause of transplant failure while advances in methods of graft-versus-host disease (GVHD) prophylaxis and supportive care have led to significant improvements in non-relapse mortality. Hence understanding the immunobiology of the graft-versus leukemia (GVL) effect and it’s perturbations resulting in relapse are paramount to improving transplant outcomes in the modern era. This review series focuses on various approaches to augment GVL, rooted in an evolving understanding of the biology of GVL and immune escape mechanisms that result in a failure of GVL and relapse. Teshima et al. review the separation of GVL from GVHD, a long-standing holy grail in the field of transplantation. They highlight attempts to enhance localized immune suppressive therapies and modulate T cell migration to better target the bone marrow niche, as well as promotion of tissue tolerance through a deeper understanding of the biology of tissue stem cells in GVHD target organs. Other avenues include counteracting immune evasion of leukemia cells, including by modulation of the tumor microenvironment. Pacini et al. look at the issue of separating GVL from GVHD specifically through the lens of regulatory T-cells or Tregs which have been traditionally associated with dampening of the GVHD response with concerned that it could also dampen the GVL effect. The authors explore strategies to enhance GVL post-Treg infusion and the proposed mechanisms for the maintenance of the GVL following adoptive Treg transfer. They further highlight refining Treg sources for infusion and evaluating their specificity for antigens mediating GVHD while preserving GVL responses. Burk et al. examine yet another aspect of GVL-the cellular metabolic underpinnings of cancer cells, immune cells and the interactions between them focusing on recent advances in the understanding of how metabolism might affect the anti-leukemia immune response. These include the recognition that leukemia and myeloid cell metabolism contribute to an altered microenvironment that impairs T cell function, the metabolic processes in AML cells are linked to their inhibitory checkpoint ligand expression and the role of systemic metabolism in GVL. A broader understanding of these aspects may eventually lead to therapies targeting cellular metabolism and augment GVL functionalities. In the remaining articles in this series, authors review various current and future interventions that can prevent or treat relapse drawing from a better understanding of immune and non-immune mechanisms of relapse. Murdock et al. highlight genomically targeted therapies and non-targeted chemotherapies that are increasingly being incorporated into pre-transplant conditioning, as post-transplant maintenance or pre-emptive therapy in the setting of mixed/falling donor chimerism or persistent detectable measurable residual disease (MRD). They explore how these emerging therapies modulate engraftment, GVHD potential, and function of the donor graft on the one hand, and how they affect the immunogenicity and sensitivity of leukemic clones to the GVL effect. Pivoting to adoptive cellular therapies, Maurer and Antin discuss current knowledge about mechanisms of GVL after donor lymphocyte infusions (DLI), experimental strategies for augmenting GVL by manipulation of DLI (e.g. neoantigen vaccination, specific cell type selection/depletion) and research outlook for improving DLI and cellular immunotherapies for hematologic malignancies through better molecular definition of the GVL effect. Hadjis et al. then review NK cell alloreactivity and their role in the GVL effect particularly in the context of PTCy based platforms. While KIR genotyping is still not routinely employed due to conflicting data and therefore does not play a role in donor selection, adoptive NK cell therapies are being used for the prevention and treatment of relapse post-transplantation including cytokine induced memory like (CIML) NK cells and the developing frontier of CAR NK cells which represents an exciting new paradigm in the post-transplant anti-leukemic armamentarium. A slightly different approach is highlighted by Rambaldi et al. in their review of modulations of adoptive T-cell therapies or traditional donor lymphocyte infusions (DLI) including cytokine-induced killer (CIK) cells. CIK cells are T lymphocytes activated in culture in the presence of monoclonal antibodies against CD3 (OKT3), interferon-gamma (IFN-g), and interleukin-2 (IL-2), characterized by the expression of markers typical of NK cells and T cells (CD3+, CD56+, with a prevalent CD8+ phenotype) and which mediate cytotoxicity through both MHC and non-MHC restricted recognition, the so‐called “dual‐functional capability” displaying minimum alloreactivity. Allogeneic CIK cells are being used to target MRD with low rates of GVHD and are also promising for adoption for building next-generation CAR therapies. Rambaldi et al. also review other modifications of DLI including selected DLI and activated DLI strategies. Overall, arising from a better understanding of the immunobiology of GVL and mechanisms leading to relapse, further optimization of adoptive T-cell and NK cell therapies and the use of targeted and non-targeted chemotherapeutic agents in the prophylactic, pre-emptive and therapeutic setting, as well as combinatorial approaches, may lead to better outcomes with post-transplantation relapse, a major endeavor in the field in the next decade.

